# Risk factors and events in the adult intensive care unit associated with pain as self-reported at the end of the intensive care unit stay

**DOI:** 10.1186/s13054-020-03396-2

**Published:** 2020-12-07

**Authors:** Pierre Kalfon, Mohamed Boucekine, Philippe Estagnasie, Marie-Agnès Geantot, Audrey Berric, Georges Simon, Bernard Floccard, Thomas Signouret, Mélanie Fromentin, Martine Nyunga, Juliette Audibert, Adel Ben Salah, Bénédicte Mauchien, Achille Sossou, Marion Venot, René Robert, Arnaud Follin, Anne Renault, Maïté Garrouste-Orgeas, Olivier Collange, Quentin Levrat, Isabelle Villard, Didier Thevenin, Julien Pottecher, René-Gilles Patrigeon, Nathalie Revel, Coralie Vigne, Elie Azoulay, Olivier Mimoz, Pascal Auquier, Karine Baumstarck, IPREA Study Group, Karine Vie, Gwenaëlle Lannuzel, Hélène Bout, Jean-Philippe Parthiot, Isabelle Chazal, Philippe Charve, Caroline Prum, Jean-Pierre Quenot, Nora Perrot, Francis Augier, Niloufar Behechti, Claudine Cocusse, Céline Foulon, Laurence Goncalves, Abdesselem Hanchi, Etienne Legros, Ana Isabel Mercier, Nicolas Meunier-Beillard, Nathalie Nuzillat, Alicia Richard, Claire Boulle, Benjamin Kowalski, Elisa Klusek, Tarek Sharshar, Andrea Polito, Caroline Duvallet, Sonia Krim, Nicolas Girard, Cécile Jourdain, Stéphane Techer, Corinne Chauvel, Corinne Bruchet, Johanna Temime, Stéphanie Beaussart, Fabienne Jarosz, Jullien Crozon-Clauzel, Serge Olousouzian, Sylvie Pereira, Loïc Argentin, Valérie Cerro, Déborah Levy, Sébastien Andre, Christophe Guervilly, Laurent Papazian, Myriam Moussa, Stéphanie Renoult, Delphine Biet, Steve Novak, Jean-Christophe Orban, Aminata Diop, Carole Ichai, Antoine Tesniere, Jean-Pascal Goupil, Frédérique Laville, Nadège Rutter, Sandie Brochon, Kelly Tiercelet, Julien Amour, Nora Ait-Hamou, Marjorie Leger, Virginie Souppart, Emilie Griffault, Marie-Line Debarre, Céline Deletage, Anne-Laure Guerin, Carole Guignon, Sabrina Seguin, Christophe Hart, Kathy Dernivoix, Caroline Wuiot, Karine Sanches, Stéphane Hecketsweiler, Catherine Sylvestre-Marconville, Vincent Gardan, Stéphanie Deparis-Dusautois, Yana Chaban

**Affiliations:** 1 Réanimation polyvalente, Hôpital Louis Pasteur, Centre Hospitalier de Chartres, Le Coudray, 28018 Chartres Cedex, France; 2 Unité de recherche EA3279, Aix-Marseille Université, Marseille, France; 3 Réanimation, Clinique Ambroise Paré, Neuilly/Seine, France; 4 Département d’Anesthésie Réanimation, CHU Dijon Bourgogne, Dijon, France; 5 Réanimation polyvalente, Centre Hospitalier Intercommunal Toulon/La Seyne sur mer, Toulon, France; 6 Réanimation, CH Troyes, Troyes, France; 7 Réanimation polyvalente, CHU Édouard Herriot, Hospices Civils de Lyon, Lyon, France; 8 Réanimation, Hôpital Européen de Marseille, Marseille, France; 9 Réanimation chirurgicale, CHU Cochin, Assistance Publique-Hôpitaux de Paris (AP-HP), Paris, France; 10 Réanimation polyvalente, CH Victor Provo, Roubaix, France; 11 Réanimation, CH Émile Roux, Le Puy-en-Velay, France; 12 Réanimation médicale, CHU Saint-Louis, AP-HP, Paris, France; 13 Réanimation médicale, CHU La Milétrie, Poitiers, France; 14 Réanimation chirurgicale, Hôpital Européen Georges Pompidou, AP-HP, Paris, France; 15 Réanimation médicale, CHU Brest, Brest, France; 16 Médecine intensive et reanimation, Groupe Hospitalier Paris Saint-Joseph, Paris, France; 17 Réanimation chirurgicale polyvalente, Hôpital Civil, CHU Strasbourg, Strasbourg, France; 18 Réanimation, Groupe Hospitalier de La Rochelle-Ré-Aunis, La Rochelle, France; 19 Anesthésie Réanimation, CHU Beaujon, AP-HP, Clichy, France; 20 Réanimation, CH Lens, Lens, France; 21 Réanimation Chirurgicale, Hôpital Hautepierre, CHU Strasbourg, Strasbourg, France; 22 Réanimation, CH Auxerre, Auxerre, France; 23 Réanimation Médico-Chirurgicale, Hôpital Pasteur, CHU Nice, Nice, France; 24 Réanimation Chirurgicale, CHU Hôpital Nord, Assistance Publique–Hôpitaux de Marseille, Marseille, France; 25 Centre Hospitalier (CH) D’Auxerre, Auxerre, France; 26 Centre Hospitalier (CHU) de Brest, Brest, France; 27 CHU Beaujon, Assistance Publique-Hôpitaux de Paris (AP-HP), Paris, France; 28 CHU Dijon Bourgogne, Dijon, France; 29 CH de Douai, Douai, France; 30 Raymond Poincaré, AP-HP, Garches, France; 31 Hospitalier de La Rochelle-Ré-Aunis, La Rochelle, France; 32 de Chartres, Chartres, France; 33 Emile Roux, Le Puy-en-Velay, France; 34 CH de Lens, Lens, France; 35 CHU Edouard Herriot, Hospices Civils de Lyon, Lyon, France; 36 Hôpital Européen de Marseille, Marseille, France; 37 CHU Hôpital Nord, Assistance Publique Hôpitaux de Marseille, Marseille, France; 38 Clinique Ambroise Paré, Neuilly/Seine, France; 39 CHU Nice, Nice, France; 40 CHU Cochin, AP-HP, Paris, France; 41 CHU Hôpital Européen Georges Pompidou, AP-HP, Paris, France; 42 Groupe Hospitalier Paris Saint-Joseph, Paris, France; 43 CHU Pitié-Salpêtrière, AP-HP, Paris, France; 44 CHU Saint-Louis, AP-HP, Paris, France; 45 CHU La Milétrie, Poitiers, Poitiers, France; 46 CH Victor Provo, Roubaix, Roubaix, France; 47 CHU Strasbourg, Strasbourg, France; 48 Centre Hospitalier Intercommunal Toulon/La Seyne sur mer, Toulon, France; 49 CH de Troyes, Troyes, France

**Keywords:** Critical care, Pain, Discomfort, IPREA, Chest drain, Intra-hospital transport, Patient-reported outcome, Intensive care unit

## Abstract

**Background:**

The short-term and long-term consequences of the most frequent painful procedures performed in the ICU are unclear. This study aimed to identify the risk factors associated with pain-related discomfort perceived by critically ill patients during the whole ICU stay as self-reported by patients at the end of their ICU stay.

**Methods:**

The study involved 34 ICUs. Adult patients who survived an ICU stay of 3 calendar days or more were eligible for inclusion. Discomforts, including the pain-related discomfort, were assessed using the French 18-item questionnaire on discomfort in ICU patients, the “Inconforts des Patients de REAnimation” (IPREA). Patients scored each item from 0 (minimal discomfort) to 10 (maximal discomfort). Associations between patient characteristics at ICU admission, life support therapies and main potentially painful procedures performed during the ICU stay and pain-related discomfort scores assessed at the end of the ICU stay were analyzed.

**Results:**

Patients with complete IPREA questionnaires (*n* = 2130) were included. The median pain-related discomfort score was 3 (IQR 0–5). From the univariate analysis, pain-related discomfort scores were negatively correlated with age and positively correlated with ICU stay duration; surgical patients reported significant higher pain-related discomfort scores than medical patients; chest drain insertion, chest drain removal, use of bladder catheter, central venous catheter (CVC) insertion, complex dressing change, and intra-hospital transport were associated with pain-related discomfort scores. From the multivariate analyses using generalized estimating equations models, only age, chest drain removal, use of a bladder catheter, CVC insertion, and intra-hospital transport were the main risk factors associated with pain-related discomfort scores.

**Conclusion:**

Patients who underwent chest drain removal, bladder catheter, CVC insertion, and intra-hospital transport during their ICU stay reported higher pain-related discomfort scores (with respect to the whole ICU stay and assessed at the end of their ICU stay) than patients who did not experience these events. This study may pave the way for further targeted studies aiming at investigating a causal link between these common procedures in the ICU and adult critically ill patients’ perceptions of their ICU stay regarding recalled pain.

*Trial Registration*: Clinicaltrials.gov Identifier NCT02442934, retrospectively registered on May 13, 2015

## Introduction

Critically ill patients in intensive care units (ICUs) are exposed to stressful conditions and experience discomfort from multiple sources, such as the environment or the treatment being provided, which depends on the organization (essentially the nurse-to-patient ratio) and the patient’s health status [1–6]. Among these discomforts, pain is one of the main sources of psychological stress during an ICU stay and after hospital discharge [7, 8]. A program for reducing ICU patients’ discomfort has resulted in adult critically ill patients reporting decreased self-perceived discomfort at the end of their ICU stay. This program effectively reduced the overall discomfort scores and the scores for each discomfort item on the questionnaire “Inconforts des Patients de REAnimation” (IPREA), except those for pain [9].

Several hypotheses may explain why the program was ineffective for reducing pain scores. First, previously implemented health policies for pain management may have previously been be prioritized over managing other discomforts. Second, measures to reduce pain reported by critically ill patients may require more time to implement, because of complexity of pain management in adult critically ill patients due to highly individual pain patterns, and unique features in the ICU such as impaired communication and altered mental status, potentially painful procedures and use of invasive devices [10]. Moreover, a consistent and effective approach to pain assessment and management may require reorganization of care and redefinition of the roles of nurses and physicians in relieving procedural pain.

Better identification of procedures that cause the pain reported at the end of ICU stays would enable developing more effective strategies against pain in the ICU. Frequent ICU-associated procedures that can cause pain include arterial blood gas sampling and tracheal suctioning in mechanically ventilated patients, which lead to more frequent arterial catheter insertions and greater care for tracheal suctioning [2, 11]. Patients’ perceptions and responses to procedural pain were assessed after common ICU procedures such as turning in bed, wound drain removal, tracheal suctioning, femoral catheter removal, central venous catheter (CVC) insertion and nonburn wound dressing changes [12]. More recently, a prospective, multicenter study of pain intensity associated with ICU procedures identified chest drain removal, wound drain removal, and arterial line insertion as being associated with greater procedural pain intensity. The study also described risk factors associated with greater procedural pain in adult ICU patients [8]. Several studies have focused on preventing pain related to chest drain removal [13]. In past studies that focused on pain experienced by critically ill patients, pain was the only discomfort assessed. Pain was often rated immediately after the procedure and sometimes before the procedure, but not at the end of the ICU stay, which would allow considering the entire ICU stay and patients’ perceptions of possible repeated painful procedures. Moreover, no previous studies have evaluated procedures such as intra-hospital transport (e.g., for computed tomography). We proposed to identify the risk factors and events that have occurred during the ICU stay associated with pain-related discomfort perceived by critically ill patients during the whole ICU stay, as self-reported by critically ill patients at the end of their ICU stay, using a global ICU-related self-perceived discomfort tool and examining a large panel of frequent pain-inducing procedures, including intra-hospital transport, chest drain insertion, and chest drain removal, from a large patient population in a multicenter prospective study.

## Methods

### Study design and centers

This study was an ancillary study of the IPREA3 study [9], a multicenter, cluster-randomized (ICU), controlled, single-blind (patient), two-parallel-group study assessing the effectiveness of a tailored multicomponent program (TMCP) for reducing self-perceived discomfort in unselected ICU patients. The research protocol was previously published elsewhere [14]. Briefly, the TMCP consisted of assessment of predetermined ICU-related self-perceived discomforts, including pain, immediate and monthly feedback to healthcare teams, and site-specific tailored interventions aimed at reducing all the ICU-related self-perceived discomforts. Among those interventions, pain-related interventions included improved pain assessment, development of protocol-driven care in the ICU according to procedures specific to each participating ICU, improved management of acute pain based on pharmacological interventions (focused on the best use of multimodal analgesia, patient-controlled analgesia, locoregional anesthesia mainly in surgical ICUs, etc.) and nonpharmacological interventions (adequate information and communication with the patient during potentially painful care, patient positioning as comfortable as possible, massages in some participating ICUs, etc.).

The effectiveness of the program was assessed from the French questionnaire on ICU-related self-perceived discomforts (Table 1) [15, 16].

**Table 1:** Questionnaire on ICU-related self-perceived discomforts (IPREA): the English version

1.	Have you experienced discomfort from *noise* (alarms, radios, telephones, conversations) during the day and/or night?
2.	Have you suffered from *too much light* in your ICU room or in the hallway, especially at night?
3.	Have you felt uncomfortable in *your bed?* (that is, is the mattress too hard or too soft, head of bed too high or not high enough, sides of bed to high or low, uncomfortable pillows?)
4.	Have you suffered from *lack of sleep* compared to your usual sleep pattern?
5.	Have you felt uncomfortable due to *thirst?*
6.	Have you felt uncomfortable due to *hunger?*
7.	Have you suffered from the *cold?*
8.	Have you suffered from the *heat?*
9.	Have you had more pain than usual for you? For example from needles, catheters, tubes, being turned or washed?
10.	Have you had discomfort from *tubes/connections to machines* (such as from IV lines, electrode connections, tubes in your throat, or oxygen mask?)
11.	Have you felt *embarrassed* or did you feel your privacy was not respected? (For example during the morning wash, changes, and review by the doctors or the medical visits?)
12.	Have you suffered from *anxiety or panic* (for example, being scared that an important piece of medical equipment may malfunction or disconnect?)
13.	Have you felt *isolated* (being alone in your room, sometimes not seeing nurses or doctors nearby) and not hearing any sounds?
14.	Did the ICU *visiting hours* (that is restricting when your family and/or friends could visit you) bother you?
15.	Have you been bothered by not having a *telephone* in the room?
16.	Did you feel you were *not informed enough* about such things as your condition, treatment plan, progress or when you would leave ICU?
17.	Did you at any time feel depressed during your stay in ICU?
18.	Did you have difficulty breathing, or feel that you were struggling for air during your ICU stay?

The study included 34 centers, all in France: medical, surgical, and mixed medical–surgical ICUs at academic tertiary care hospitals or community hospitals.

### Patient sample and design

All patients aged 18 years or older who survived an ICU stay of 3 calendar days or more were eligible for inclusion. We excluded patients who died during the ICU stay, patients under trusteeship, patients whose diminished mental capacity (as assessed by the bedside ICU nurse at her discretion) prevented the administration of the IPREA questionnaire, patients who did not understand French sufficiently to answer the questions, and patients transferred to another ICU while mechanically ventilated. During the IPREA3 study, all 34 ICUs enrolled patients during three 1-month periods (at baseline and 6- and 12-month post-baseline), respectively, in October 2014, April 2015, and October 2015. The patient sample for this study comprised all patients included in the IPREA3 study during these three periods, regardless of the group, control or interventional, resulting from the randomization of the ICUs.

### Measurement of pain-related discomfort scores

The pain-related discomfort score, used as the dependent variable, is derived from the IPREA questionnaire and corresponded to item 9 and the following question: *“Have you had more pain than usual for you? For example from needles, catheters, tubes, being turned or washed*”. As each of the discomfort items, the pain-related discomfort was scored from 0 (minimal discomfort) to 10 (maximal discomfort) [16]. Each participating ICU was supplied with tablets with Internet connection if the patients’ rooms lacked computers or Internet access. On the day of ICU discharge, the bedside nurse (or assistant nurse) electronically administered the IPREA questionnaire, clearly explaining to each patient that they needed to assess each discomfort item that they experienced during their entire ICU stay to the best of the patient’s memory and not just on the day the question was asked. To ensure that the nursing staff were adequately trained, the application was used for a training period in each ICU, with the coordination team of the IPREA3 study providing technical and educational support. The order in which the discomfort items were asked was randomized to reduce corruption between items, i.e., not to influence the answer to a question by the order in which this question was asked, as it had been done during the validation study [15]. The randomization process assigned each discomfort item the same weight and did not prioritize some discomfort items over others. Thus, the question concerning pain was asked in a variable order for each patient and could be preceded by any of the other 17 questions used to assess the IPREA items.

### Ethics, consent, and permissions

Regulatory monitoring was performed in accordance with the French law requiring the approval of the French Ethics Committee (Comité de Protection des Personnes Tours region Centre-Ouest 1, 28/08//2013, reference number 2013-S10). All records and patient identities remained confidential in accordance with the regulations of the French National Committee of Informatics and Liberties (Commission nationale de l’informatique et des libertés, 20/03/2014, reference number DR-2014-097) and the French Consultative Committee for data processing in health research (Comité consultatif sur le traitement de l’information en matière de recherche dans le domaine de la santé, 12/12/2013, reference number 13.642bis). Consent was obtained from all participants.

### Data collection

Demographic and clinical characteristics were collected from patients at admission or during their first day in the ICU, including age, sex, first 24-h Simplified Acute Physiology Score (SAPS II), scores exploring prior health status (i.e., Knaus score [17]), reason for ICU admission (medical vs. surgical), location before the ICU, and category of main diagnosis at admission. The total number of days in the ICU and life support therapies performed during the ICU stay to treat main organ failure (i.e., mechanical ventilation [MV], noninvasive ventilation [NIV], use of vasopressors, and renal replacement therapy) were recorded. Presence of the main potentially painful or discomfort-inducing procedures (i.e., chest drain insertion, chest drain removal, use of bladder catheter, arterial catheter insertion, CVC insertion, bronchoscopy, complex dressing change, and intra-hospital transport) during the ICU stay was recorded. For each of these procedures, the ventilatory status (performed under spontaneous ventilation, NIV, or MV) was collected.

### Statistical analysis

Sample characteristics were described using numbers and percentages, mean and standard deviation (SD), and median and interquartile range (IQR). Pain-related discomfort scores were compared between subgroups (sex [18, 19], patient type, Knaus score, invasive procedures performed during the ICU stay, group i.e., hospitalization in an ICU either applying the tailored multicomponent program to reduce discomfort in the ICU for 5 months or having never applied the program and month during which the patient was discharged from the ICU, April vs. October) using Mann–Whitney tests; the links between pain scores and continuous variables (age [20], SAPS II, ICU stay duration) were assessed using Spearman’s correlation coefficients. Multivariate analyses using generalized estimating equations models were performed to identify variables linked to self-reported pain-related discomfort scores. Independent variables relevant to the models were selected from the univariate analysis based on a threshold *P* value of ≤ 0.10, and with potential interest for adjustment (group and inclusion month). The center was used as within-subject effect. The final models produced beta coefficients and standard errors. Independent variables with higher beta coefficients are those with greater relative effect on pain-related discomfort score. Statistical analyses were performed in accordance with the statistical analysis plan using SPSS software (IBM SPSS PASW Statistics Inc., Chicago, IL USA). All tests were two-sided. *P* < 0.05 was considered statistically significant.

## Results

### ICUs and patients

Among the 34 ICUs participating in the IPREA3 study, 12 were surgical ICUs (including three cardiac surgical ICUs), seven were medical ICUs, and 15 were mixed (medical and surgical) ICUs. Twenty-two of the 34 ICUs were located at tertiary care hospitals; 12 were at community hospitals. During the three 1-month enrollment periods of the IPREA3 study, 2130 patients were included; 634, 758, and 738 were included in November 2014, April 2015, and November 2015, respectively. Table 2 details the main demographic and clinical characteristics. The patients were mostly men, with a mean age < 65 years. According to the Knaus score, almost half the patients were defined as having moderate limitations, and 29% were defined as having a severe limitation. The reasons for ICU admission were medical for 48% and surgical for 52% of patients. Respiratory was the most frequent category (28%) of main diagnoses among medical patients, and cardiovascular surgery was the most frequent surgery (20%) among surgical patients. Table 3 presents the ICU stay duration, life support therapies, and main invasive procedures performed during the ICU stay.

**Table 2:** Patients’ demographic and clinical characteristics at admission (*n* = 2130 patients)

Variable	
Age, year; mean (SD)	63 (16)
Male sex, *n* (%)	1388 (65%)
SAPS II score^a^, mean (SD)	36.4 (16.6)
Knaus score^b^, *n* (%)	
Normal health status	458 (22%)
Moderate activity limitation	1024 (48%)
Severe activity limitation due to chronic disease	613 (29%)
Bedridden patient	35 (2%)
Reason for ICU admission^b^, *n* (%)	
Medical	1017 (48%)
Surgical (scheduled)	723 (34%)
Surgical (emergency)	390 (18%)
Location before admission^b^, *n* (%)	
Emergency department	701 (33%)
Operating theater or postoperative recovery room	863 (41%)
Hospital floor (or ward)	386 (18%)
Another ICU	71 (3%)
Intermediate care unit	41 (2%)
Other	68 (3%)
Category of main diagnosis at admission^b^, *n* (%)	
Respiratory	429 (28%)
Cardiovascular	125 (6%)
Gastrointestinal or liver	116 (5%)
Renal	100 (5%)
Neurologic	105 (5%)
Hematologic or oncologic	26 (1%)
Metabolic	45 (2%)
Other medical category	161 (8%)
Gastrointestinal or urological surgery	298 (14%)
Cardiovascular surgery with ECC	342 (16%)
Cardiovascular surgery without ECC	83 (4%)
Neurosurgery	36 (2%)
Orthopedic surgery	75 (4%)
Thoracic surgery	99 (5%)
Other surgical category	90 (4%)

*SAPS* Simplified Acute Physiology Score, *ICU* intensive care unit, *ECC* extracorporeal circulation

^a^SAPS II score may range from 0 to 156, with higher scores indicating more severe illness

^b^Percentages might not total 100% because of rounding

**Table 3:** ICU stay duration, life support therapies, and procedures during patients’ ICU stays (*n* = 2130 patients)

Variable	
Days in ICU, median (interquartile range)	5 (3–8)
Life support therapies	
Mechanical ventilation, *n* (%)	1203 (56%)
Noninvasive ventilation, *n* (%)	712 (33%)
Use of vasopressors, *n* (%)	867 (41%)
Renal replacement therapy, *n* (%)	182 (9%)
Procedures during ICU stay, *n* (%) (total involved patients)	
Chest drain insertion	150 (7%)
Chest drain removal	398 (19%)
Use of bladder catheter	1763 (83%)
Arterial catheter insertion	886 (42%)
Central venous catheter insertion	789 (37%)
Bronchoscopy	176 (8%)
Complex dressing change	419 (20%)
Intra-hospital transport	610 (29%)

*ICU* intensive care unit

^a^Use of bladder catheter during any period of the ICU stay was recorded without information on patients’ ventilation conditions (mechanical ventilation, NIV, or SV) at the time of bladder catheter insertion

### Variables associated with pain-related discomfort score

The median pain-related discomfort score was 3 (IQR 0–5). From the univariate analysis, age was negatively correlated with pain-related discomfort score and positively correlated with the ICU stay duration. Medical patients reported less pain-related discomfort than did surgical patients, and patients treated with MV during their stay reported greater pain-related discomfort than did other patients (Table 4). Patients who experienced chest drain insertion, chest drain removal, bladder catheter, CVC insertion, complex dressing changes, or intra-hospital transport reported more pain-related discomfort than did other patients. Multivariate analysis showed that higher pain-related discomfort was associated with younger age, chest drain removal, bladder catheter use, CVC insertion, and intra-hospital transport (Table 5).

**Table 4:** Determinants of pain-related discomfort scores: univariate analysis (*n* = 2130 patients)

			*P* value
Sex	Men	3 (0–5)	0.556
	Women	3 (0–5)	
**Age**	*R*	− 0.051	**0.019**
Patient type			
**Medical vs. surgical**	Medical	2 (0–5)	**0.007**
	Surgical	3 (0–5)	
Cardiovascular and thoracic surgical patients	No	2 (0–5)	0.904
	Yes	3 (0–5)	
Cardiovascular surgical patients	No	2 (0–5)	0.477
	Yes	3 (0–5)	
Thoracic surgical patients	No	3 (0–5)	0.269
	Yes	2 (0–4)	
Knaus score	Normal	3 (0–5)	0.127
	Moderate	3 (0–5)	
	Severe	2 (0–5)	
SAPS II	*R*	0.006	0.770
**ICU stay duration**	*R*	0.046	**0.034**
Life support therapies during ICU stay			
**Mechanical ventilation**	No	2 (0–5)	**0.051**
	Yes	3 (0–5)	
Noninvasive ventilation	No	3 (0–5)	0.404
	Yes	2.5 (0–5)	
Vasopressor administration	No	3 (0–5)	0.425
	Yes	2 (0–5)	
Renal replacement therapy	No	3 (0–5)	0.157
	Yes	3 (0–5)	
Painful or discomfort-inducing invasive procedures during ICU stay			
**Chest drain insertion**	No	2 (0–5)	**0.037**
	Yes	3 (1–5)	
**Chest drain removal**	No	2 (0–5)	**< 0.001**
	Yes	3 (1–5)	
**Use of bladder catheter**	No	2 (0–5)	**0.001**
	Yes	3 (0–5)	
Arterial catheter insertion	No	3 (0–5)	0.555
	Yes	2.5 (0–5)	
**Central venous catheter insertion**	No	2 (0–5)	**0.014**
	Yes	3 (0–5)	
Bronchoscopy	No	3 (0–5)	0.351
	Yes	3 (0–5)	
**Complex dressing change**	No	2 (0–5)	**0.032**
	Yes	3 (0–6)	
**Intra-hospital transport**	No	2 (0–5)	**< 0.001**
	Yes	3 (0–5)	
Program	Never applied	2 (0–5)	0.754
	Applied for 5 months or more	3 (0–5)	
Inclusion month	April	3 (0–5)	0.314
	October	3 (0–5)	

*ICU* intensive care unit, *SAPS* Simplified Acute Physiology Score,* R* Spearman’s correlation coefficient

Data are presented as the median with interquartile range (IQR) in parentheses

Bold font highlights statistically potential determinants of pain-related discomfort scores as self-reported at the end of patients’ ICU stay (*P* value ≤ 0.10)

**Table 5:** Determinants of pain-related discomfort scores: multivariate analysis (*n* = 2130 patients)

	Beta (SE)	*p* value
**Age**	**− 0.013 (0.004)**	**0.002**
Patient type (medical vs. surgical)	0.013 (0.160)	0.417
ICU stay duration	− 0.001 (0.008)	0.937
Mechanical ventilation	− 0.142 (0.154	0.357
Chest drain insertion	0.017 (0.284)	0.951
**Chest drain removal**	**0.477 (0.177)**	**0.007**
**Use of bladder catheter**	**0.394 (0.187)**	**0.035**
**Central venous catheter insertion**	**0.314 (0.155)**	**0.043**
Complex dressing change	0.196 (0.176)	0.265
**Intra-hospital transport**	**0.381 (0.155)**	**0.014**
Program (never applied vs. applied for 5 months or more	0.06 (0.127)	0.640
Inclusion month (April vs. October)	0.165 (0.132)	0.210

Data are presented as the median with interquartile range (IQR) in parentheses

*SE* standard errors

Bold font highlights statistically potential determinants of pain-related discomfort scores as self-reported at the end of patients’ ICU stay (*P* value ≤ 0.10)

## Discussion

We used a large sample of 2130 French ICU patients treated at academic tertiary care and community hospitals to determine factors modulating pain-related discomfort during ICU stays. All the patients were previously included in the IPREA3 study, whose primary endpoint was the overall discomfort score derived from the French 16-item IPREA questionnaire. This first version of the IPREA questionnaire was validated (several years before the start of the IPREA3 study) through a process based on international guidelines using a large sample of critically ill patients hospitalized in different ICU types (medical, surgical, and mixed ICUs) at different institutions (university and nonuniversity hospitals) [15]. However, during the IPREA3 study, patients were questioned with a 18-item version of the IPREA questionnaire resulting from the addition of two discomfort items, item 17 and item 18 corresponding to dyspnea and feeling depressed, respectively, to the previous 16-item version. Since then, an ancillary study of the IPREA3 study yielded the psychometric validation of the 18-item version of the IPREA questionnaire [16].

To our knowledge, this was the first study exploring the weight of a large panel of potentially pain- or discomfort-inducing procedures or treatments used to treat critically ill patients.

The four procedures undergone by patients during the ICU stay that were associated with greater pain-related discomfort were chest drain removal, bladder catheter use, CVC insertion, and intra-hospital transport.

Chest drain-related procedures (insertion or removal) seemed to more strongly affect pain perception during ICU stays than did most other procedures considered invasive or discomfort-inducing (e.g., arterial catheter insertion or bronchoscopy). It is also important to note that no specific mandatory measure to reduce pain associated with either chest drain insertion or chest drain removal has been implemented during the study period in the participating ICUs. Previous studies that found chest drain-related procedures to be painful [21, 22] often did not differentiate between insertion and removal times. We showed that the two procedures did not induce the same pain-related discomfort: chest drain removal appeared to be more painful than chest drain insertion. This information may help intensivists decide the timing of analgesics prior to removing a chest drain [23]. Greater attention should be given to pain relief before and during this procedure, leading to the use of previously described nonpharmacological methods [24–26]. Of note, critically ill surgical patients may be admitted to the ICU with a chest drain having been previously inserted in the operating room under general anesthesia and therefore will not require this procedure in the ICU. Thus, chest drain removal is more common than chest drain insertion during ICU stays. However, chest drains may be left in place during transfers from the ICU to the hospital surgical ward. Because of the large number of surgical patients, the number of patients who underwent at least one chest drain removal during their ICU stay was more than double that of patients who had at least one chest drain inserted. Remarkably, of the 2130 patients included here, many more patients underwent at least one chest drain removal under spontaneous ventilation or NIV than under MV.

Intra-hospital transport during ICU stays was also associated with self-reported pain-related discomfort. Intra-hospital transport of critically ill patients is a well-known risk factor for unexpected adverse events and complications, such as pulmonary complications, hemodynamic complications, infection, interruption of critical drugs, etc. [27–31], but an association between intra-hospital transport and higher pain-related discomfort scores being reported at the end of the ICU stay is surprising. We did not record the reasons for intra-hospital transport. We hypothesize that most patients were transferred to the radiology department for computed tomography scans, and moving patients from the critical care bed to the examination table may be comparable to turning them in bed, which was previously reported by critically ill patients to be one of the most painful procedures [12, 32].

As previously reported, younger people were also independently associated with higher self-reported pain-related discomfort scores compared with those of older people [33].

Sex, patient type (i.e., medical vs. surgical), prior health status, and ICU stay duration were not independently associated with pain scores.

We used a comprehensive approach because the patients interviewed at the end of the ICU stay were not specifically informed of precisely what happened to them during their ICU stay before the discomfort assessment. Moreover, the interviewed patients were not asked if they remembered having undergone a particular procedure but only to score each of the 18 discomfort items. For example, patients may be unaware of having a chest drain inserted or withdrawn, a bladder catheter, a CVC insertion or being transported outside the ICU at any time during their stay. The healthcare team did not control or influence patients’ memories, thus reducing any suggestive bias. Moreover, the patients were not interviewed immediately after the potentially painful event as is the case in most studies aiming to assess or prevent procedural pain [34].

Our study had several limitations. First, even if the bedside nurse asked patients to rate the severity of each discomfort for the entire stay in the ICU, patients may have reported discomforts, including pain, experienced only during the final part of the ICU stay if they could not remember what they experienced at the beginning of their ICU stay. Thus, events occurring during the last days of their ICU stay, such as chest drain removal after the clinical situation improved, may have had more influence on the pain-related discomfort score than did events that occurred at the beginning of their ICU stay, such as arterial catheter insertion or bronchoscopies. Future studies should record and analyze the time course of all procedures and life support therapies undertaken during the ICU stay. Second, all discomfort item scores, particularly the pain-related discomfort scores, were obtained with the help of a nonblinded bedside nurse (or assistant nurse), which could introduce bias. We accepted that conducting a pragmatic trial to explore potential benefits in the ICU with such an easily reproducible program would require participation from bedside nurses. Third, we could not assess the link between potential painful or discomfort-inducing procedures or life support therapies and the overall ICU-induced pain burden experienced in nonsurvivors during their ICU stay because ICU nurses administered the IPREA only to survivors on the day of discharge. This limitation also applied to patients who could not be questioned such as those with diminished mental capacity, those with language barriers, those who were transferred to another ICU while mechanically ventilated, or those who were urgently discharged. The issue of representativeness of this sample of ICU survivors among the general population of critically ill patients remains uncertain. Fourth, we could not analyze the impact of analgesic or sedative drug dosages or nonpharmacological therapies used during the procedures or the implementation of life support therapies. Fifth, we did not record details concerning chest drain-related procedures [35, 36]. Drain size (e.g., indwelling small catheter, small-bore chest drain, large-bore chest drain), use of premedication and/or local anesthesia, confirmation of the insertion site via ultrasonography or needle suction, chest wall dissection (by finger, curved hemostat or clamp, or trocar), and parietal refection and sutures (none, “mattress” suture, or “purse string” suture) would enable better understanding the self-perceived pain. However, the goal of the study was not to determine the exact role of any factor characterizing each potential painful or discomfort-inducing procedure nor was it to propose recommendations for preventing pain or discomfort when performing these procedures. Based on the standard procedures and treatments used in the ICU, our study constituted a first approach to detect those likely to remember pain during their ICU stay to design further studies to understand the involved mechanisms in greater detail. Sixth, recent recommendations on variable selection have been proposed [37]. However, we assumed that our approach to identify variables associated with recalled pain, based on consensual and well-recognized selection procedure, remains robust and valid. Finally, we acknowledged that our study was not designed to assess and distinguish recalled pain associated with procedures from recalled pain not associated with them. We only sought an association of the achievement of some commonly used procedures in the ICU with pain experienced during the entire ICU stay as it was self-reported at the end of the ICU stay, whether pain was exacerbated—or caused—by procedures or whether pain was already present between the procedures due to other causes.

However, despite these limitations and without being able to affirm the causal link between these events and the painful memorization of the ICU stay at discharge, these first results showing an association between the occurrence of such potentially painful procedures and the pain-related discomfort score should encourage us to properly assess the balance of the benefits and risks of these common procedures such as maintenance of a bladder catheter, intra-hospital transport for an iterative CT scan, CVC insertion or thoracic drainage.

## Conclusion

Our study showed that adult ICU patients who underwent chest drain removal, bladder catheter, CVC insertion, and intra-hospital transport during their ICU stay reported higher pain-related discomfort scores (regarding the entire ICU stay and assessed at the end of the ICU stay) than patients who did not experience these events. This study may pave the way for further targeted studies aiming at investigating a causal link between these common procedures undergone by adult critically ill patients during their ICU stay and recalled pain at ICU discharge.

## Data Availability

The datasets used and analyzed during the current study are available from the corresponding author in response to reasonable requests.
